# Transradial Coronary Intervention in Chronic Total Occlusion in a Patient With Mirror Image Dextrocardia

**DOI:** 10.7759/cureus.13991

**Published:** 2021-03-19

**Authors:** Anupam Bhambhani, Amey Joshi

**Affiliations:** 1 Cardiology, Vydehi Institute of Medical Sciences and Research Centre, Bangalore, IND

**Keywords:** dextrocardia, percutaneous coronary intervention, radial access, chronic total occlusion, situs-inversus-totalis

## Abstract

Dextrocardia poses challenges in the percutaneous coronary intervention, particularly through radial access. The presence of chronic total occlusion further adds to the technical difficulties in such cases due to unfamiliar orientations of the coronary arteries, guide catheter instability, and problems in advancing the hardware across the occluded lesions.

We report here a successful percutaneous intervention in a chronic total occlusion of the left anterior descending coronary artery, done through right radial access in a patient with situs-inversus and dextrocardia.

The trans-radial percutaneous intervention approach is safe and feasible in patients with dextrocardia. Pre-planned imaging strategies and the choice of appropriate hardware tremendously help in successfully completing the intervention in such cases.

## Introduction

Percutaneous coronary intervention (PCI) through trans-radial access (TRA) is favored by the interventional cardiologists across the globe, but the presence of situs-inversus-totalis makes it challenging, mainly due to the difficulties encountered in achieving co-axial positioning of the guide catheters. Right TRA further alters the catheter course at the subclavian artery take-off from the aortic arch because of the unusual angular relationship between the two vessels.

We report here a successful PCI in chronic total occlusion (CTO) of the left anterior descending (LAD) coronary artery, done through right TRA, in a patient with situs-inversus and dextrocardia.

## Case presentation

**Figure 1:**
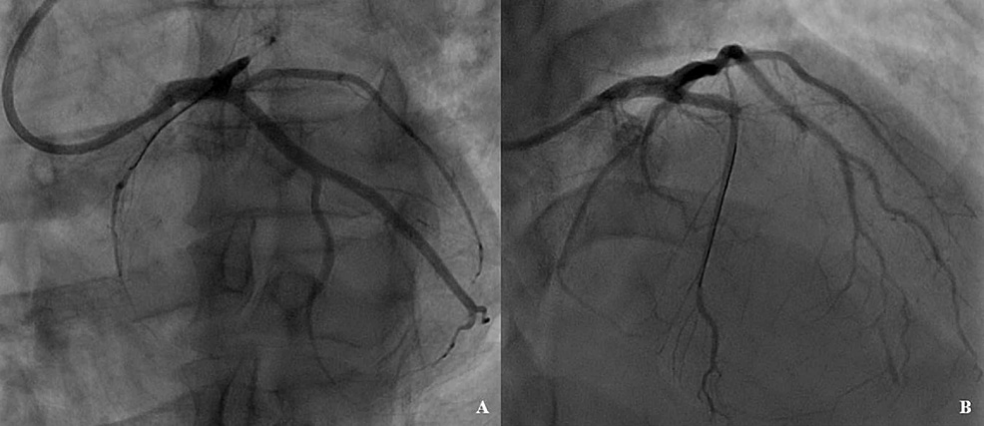
Left coronary angiograms obtained after applying digital left-right image inversion, while the camera was positioned at horizontally reverse angles (double inversion). (A) Caudally tilted right anterior oblique image resembling conventional spider view. (B) Cranially tilted left anterior oblique image resembling conventional right anterior oblique view. Guidewire in septal artery, serving as anchor, can be seen in both these pictures.

**Figure 2:**
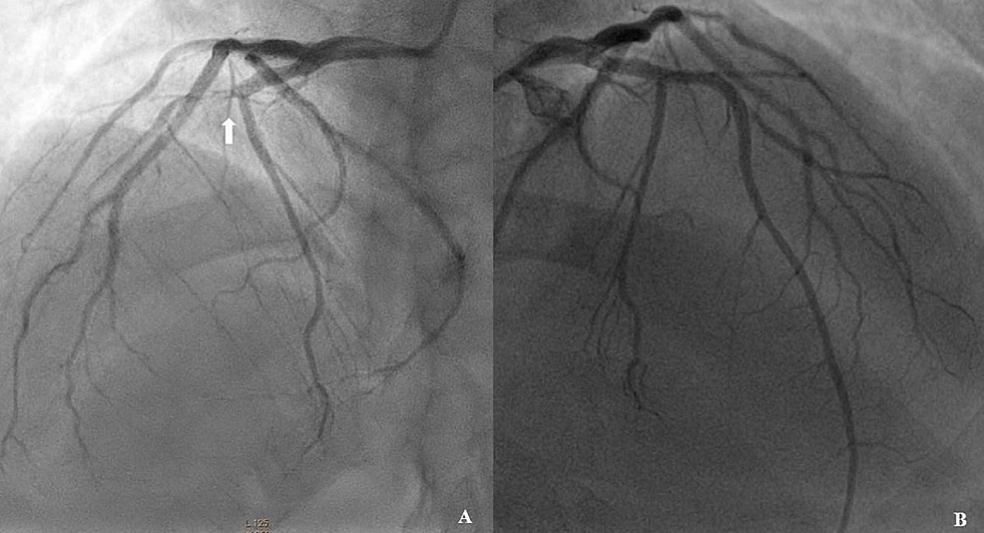
Left coronary angiograms before (A) and after (B) the intervention (arrow is pointing at the chronic total occlusion in left anterior descending artery).

A 59-year-old gentleman, a known case of mirror-image dextrocardia, presented with chronic stable angina that was partially responsive to medical therapy. The pain was retro-sternal and radiated to the right shoulder. There were no conventional risk factors for ischemic heart disease. A 12-lead electrocardiogram was consistent with dextrocardia and was otherwise unremarkable. Coronary angiography was done through the right radial access using 5F TIG- Optitorque® catheter (Terumo-interventional-systems, Tokyo, Japan). It demonstrated a 100% stenosis in the mid-segment of LAD with Rentrop grade 2 collaterals (Figures 1A, 1B, 2A, Video 1). There was no significant stenosis in other coronary arteries.

**Video 1:** Left coronary angiogram showing 100% stenosis in LAD with Rentrop grade 2 collaterals. LAD - left anterior descending

We decided to perform PCI to LAD through right radial access. Cannulation of the left coronary artery (LCA), situated on the right side in this case, was attempted using a 6F 3.5 curve Extra-backup coronary guide-catheter (Medtronic Launcher, Santa Rosa, CA, USA) by maneuvering it with counter directional rotations, i.e. wherever clockwise rotation works in normal situs, the counter-clock rotation was performed and vice versa. This method failed to position the catheter selectively into the coronary ostium. In order to achieve selective LCA cannulation, the catheter was advanced into the respective sinus with counter-clock rotation and then was pushed up from the aortic root with simultaneous clockwise rotation. This maneuver positioned the catheter in the aortic sinus in a way that it faced the LCA ostium (Video 2).

**Video 2:** Non-selective cannulation of left coronary artery.

Following this, a 0.14 inch-180 cm Sion Blue (ASAHI-INTECC, USA) guidewire was advanced into the septal artery and used as an anchor, over which the catheter could be advanced to a desirable coaxial position into the LCA ostium (Video 3).

**Video 3:** Selective cannulation of coronary artery achieved with the help of anchor wire in septal branch of left anterior descending artery.

In order to make the CTO anatomy clearer and to achieve vessel orientation closer to normal, we acquired further images with right-left image inversion on the screen (by digitally activating horizontal reverse sweep on the console) and, at the same time, reversed the horizontal angles of image-intensifier (camera) position, while maintaining the conventional cranial-caudal angles. In this way, the camera position in caudally tilted right-anterior-oblique (RAO) angle provided coronary image resembling spider view (Figure 1A), while in cranially tilted left-anterior-oblique angle, it provided coronary image resembling routine RAO-cranial view (Figure 1B). By practicing this double inversion technique, angiographic images resembling normal situs were obtained.

After adequate imaging, crossing of the CTO was attempted using another 0.014 inch-180 cm Sion Blue wire. The initial failure of this wire necessitated the support of a 1.25x6 mm semi-compliant balloon, using which the proximal cap of the CTO could be penetrated (Video 4).

**Video 4:** Penetrating proximal cap of the chronic total occlusion while stabilizing the guide catheter with help of anchor wire in the septal branch of left anterior descending artery.

While traversing the CTO body, the wire entered a large diagonal branch; leaving this wire in the diagonal branch, we retracted the first wire that was stationed in the septal branch as an anchor and advanced it into the distal LAD. During this, the wire in the diagonal branch served as an anchor for guide-catheter stability and at the same time guided us to the distal LAD like a parallel wire (Video 5).

**Video 5:** Occlusion crossed while maintaining anchor in the diagonal branch of Left Anterior Descending artery.

After serial pre-dilatations with 1.25 and 2.5 mm diameter semi-compliant balloons, a 2.75x16 mm Everolimus eluting stent was deployed across the lesion at 12 atm pressure. A final angiogram revealed a well-deployed stent with TIMI-III flow and no residual dissection (Figure 2B). The recovery was uneventful and the patient is free of angina at 15 months’ follow-up.

## Discussion

Dextrocardia poses challenges in the diagnosis as well as invasive treatment of coronary artery disease due to several factors including unusual site of anginal pain on the right side, altered QRS morphology resulting from a reverse sequence of ventricular depolarisation, unfamiliar orientations of the coronary artery branches, and technical difficulties encountered during the intervention.

Trans-radial PCI in patients with situs-inversus and dextrocardia is technically demanding due to difficulties encountered in achieving the coaxial position of conventional guide catheters. Right TRA further alters the catheter course at the subclavian artery take-off from the aortic arch, as the angular relationship between the two vessels is a mirror image of what is expected in situs-solitus. The presence of CTO in such cases adds to the complexity of the intervention due to difficulties envisaged in the angiographic assessment of the anatomy of the occluded segment, assessment of collateral channels, and in hardware advancement across the CTO, particularly with suboptimal catheter stability.

In order to review the techniques used by other operators in successfully performing PCI through TRA in the presence of mirror-image dextrocardia, we searched the databases of PubMed, PubMed Central, Google Scholar, and Cochrane for similar case reports. A total of 11 publications [1-11] were identified (Table 1), reporting PCI on 17 lesions in 13 patients.

**Table 1:** Technical details in reported cases of trans-radial coronary interventions in patients with situs-inversus and dextrocardia. TRA, transradial access; LAD, left anterior descending; RCA, right coronary artery; LCx, left circumflex

S no.	Reference	Clinical presentation and duration of symptoms	Vascular Access	PTCA guidewire used	Culprit artery and severity of stenosis	Diagnostic or guide catheter		Left-Right screen image inversion technique	Left-Right reversal of camera positioning	Special adjuvant technique
						Tried and failed	Successfully used			
1	Macdonald et al., 2007 [1]	Stable ischemic heart disease; >1 month	Right TRA	Traverse wire	Mid-segment LAD 100%	Judkin’s Left 4 and 5 curves	Extra-backup (EBU) 3.5	Not applied	Applied	Balloon support
2	Zhao et al., 2010 [2]	Acute coronary syndrome 1-day	Right TRA	Not reported	Proximal RCA 80%	None	Judkin's Right	Not applied	Not applied	None
					Mid-segment LCX 85%	None	Judkin's Left			
3	Ishiguro et al., 2011 [3]	Acute coronary syndrome 2-weeks	Right TRA	Runthrough, Grandslam guidewire	Ostial RCA Critical stenosis	Judkin’s Left-4	Diagnostic - heat modified Judkin’s Right-4; Guide catheter - Ikari Left 3.0	Not applied	Not applied	Parallel wire
4	Menozzi et al. 2012 [4]									
	Case 1	Non-ST elevation myocardial infarction; 48-hours	Right TRA	BMW	Ostial LAD 100%	Optitorque® TIGER	Extra-backup (EBU) 3.5	Not applied	Applied	None
	Case 2	ST-elevation myocardial infarction	Right TRA	BMW	Distal RCA critical stenosis	None	Judkin’s Right 4.0	Not applied	Applied	None
5	Showkathalli et al., 2012 [5]	ST-elevation myocardial infarction; (facilitated PCI)	Right TRA	Not reported	Mid and distal RCA calcific stenosis	None	Amplatz Right-2 guide catheter	Not applied	Not applied	Rotational atherectomy
6	Goel and Moorthy, 2013 [6]	ST-elevation myocardial infarction; 4-hours	Right TRA	BMW	Mid RCA 99%	None	ECR® guide catheter	Applied	Applied	None
7	Sinha et al., 2015 [7]	Unstable angina two-days	Right TRA	BMW	Mid-segment RCA 99%	None	Judkins Right-4 guide catheter	Not applied	Applied	None
				BMW	Proximal LCX 80%	Optitorque® TIGER, Judkins Left-4	Amplatz Left-2 guide catheter	Not applied	Applied	None
8	Michas et al., 2016 [8]	ST-elevation myocardial infarction; 30-minutes	Right TRA	BMW	Postero-lateral branch of RCA 99%	None	Judkins Right	Not applied	Applied	
9	Potdar et al., 2016 [9]	Unstable angina; 5-days	Right TRA	Whisper MS guide wire	Proximal RCA 80%	Judkin’s Right-4 guide catheter	Extra-backup (EBU) 3.0	Not applied	Applied	
					Mid-segment LAD 80%	None	Extra-backup (EBU) 3.0			
					Proximal segment LCX 80%	None	Extra-backup (EBU) 3.0			
10	He et al., 2016 [10]									
	Case 1	ST-elevation myocardial infarction; 1-day	Right TRA	Runthrough	Proximal LCX 100%	Optitorque® TIGER	Judkins Left 4.0, and Judkins Right 4.0 for CAG; 6F Brachial Left (BL) 3.0 Guide catheter	Not applied	Not mentioned	Thrombus aspiration
	Case 2	ST-elevation myocardial infarction; 1-hour	Right TRA	Runthrough	Proximal LAD 100%	None	6F Brachial Left (BL) 3.0 Guide catheter	Not applied	Not mentioned	None
11	Huang et al., 2020 [11]	ST-elevation myocardial infarction; 2-hours	Right TRA	Not reported	LCX 100%	Not reported	Judkins Left 4.0	Not applied	Not applied	None

Five successful PCIs were reported on occluded vessels [1,4,10,11]. Four of these interventions were done on acute occlusions of ≤24-hour duration [4,10,11], while in the remaining one, the clinical diagnosis was stable ischemic heart disease (IHD), the authors did not specify if the lesion was a CTO [1].

The earliest case of successful trans-radial PCI in dextrocardia was described by Macdonald et al. in 2004 [1]. Goel and Moorthy were the first to describe the usefulness of the double image inversion technique in dextrocardia [6], which we also used in our case. Although no other operator reported use of the double inversion, six operators described the ease of doing PCI by horizontally reversed positioning of the camera [1,4,6-9] and in one report [10], the imaging details were not mentioned. Back-up support catheters were preferred in all cases and EBU catheter, with counter direction rotations, successfully cannulated LCA in most cases. As seen in Table 1, hydrophilic-coated guidewires were preferred by most operators; however, hydrophobic wire with balloon support was used in one case [1].

Based on our and other operators’ experiences, we found the following technical steps helpful in successfully completing CTO PCI through TRA in dextrocardia patients: (a) double inversion modification while imaging, i.e. use of right-left image inversion in combination with camera positioning at horizontally opposite angles, substantially improves the ease of angiographic interpretation; (b) back-up catheters should always be preferred; in case of encountering difficulty in coaxial catheter positioning, support techniques (e.g. wire anchor or balloon anchor) should be employed instead of switching to catheters with a sub-optimal back-up; (c) counter directional rotation of catheters is instrumental in cannulation of coronary ostia; (d) extension catheters like Guideliner™ or Guidezilla™ may provide extra support if needed; (e) lubricious wires with soft tips are good for approaching the proximal cap, but need balloon or microcatheter support for penetrating and traversing the CTO.

## Conclusions

Right transradial access for PCI in dextrocardia is safe and practical. The knowledge of the atypical presentation of angina pectoris and other difficulties associated with the diagnosis of IHD in dextrocardia patients may help in the early identification and management of the disease. Pre-planned imaging strategies and appropriate choice of hardware tremendously help in successfully completing PCI in such cases.
